# A Pilot Study of Changes in the Level of Catecholamines and the Activity of α-2-Macroglobulin in the Tear Fluid of Patients with Parkinson’s Disease and Parkinsonian Mice

**DOI:** 10.3390/ijms22094736

**Published:** 2021-04-29

**Authors:** Vsevolod Bogdanov, Alexander Kim, Marina Nodel, Tatiana Pavlenko, Ekaterina Pavlova, Victor Blokhin, Natalia Chesnokova, Michael Ugrumov

**Affiliations:** 1 Koltzov Institute of Developmental Biology of the Russian Academy of Sciences, 26 Vavilova Street, 119334 Moscow, Russia; vse-bogd@yandex.ru (V.B.); alexandrrkim@gmail.com (A.K.); guchia@gmail.com (E.P.); victor.blokhin@hotmail.com (V.B.); 2 Sechenov First Moscow State Medical University of the Ministry of Health of the Russian Federation, 8/2 Trubetskaya Street, 119991 Moscow, Russia; nodell_m@yahoo.com; 3 Russian Clinical and Research Center of Gerontology, 16 1st Leonova Street, 129226 Moscow, Russia; 4 Helmholtz Moscow Research Institute of Eye Diseases of the Ministry of Health of the Russian Federation, 14/19 Sadovaya-Chernogryazskaya Street, 105062 Moscow, Russia; tanya1975_@inbox.ru (T.P.); nchesnokova2012@yandex.ru (N.C.)

**Keywords:** Parkinson’s disease, tear fluid, patients, mice, MPTP, biomarkers, lacrimal glands, catecholamines, α-2-macroglobulin

## Abstract

Development of differential and early (preclinical) diagnostics of Parkinson’s disease (PD) is among the priorities in neuroscience. We searched for changes in the level of catecholamines and α-2-macroglobulin activity in the tear fluid (TF) in PD patients at an early clinical stage. It was shown that TF in patients is characterized by an increased level of noradrenaline mainly on the ipsilateral side of pronounced motor symptoms (72%, *p* = 0.049), a decreased level of adrenaline on both sides (ipsilateral—53%, *p* = 0.004; contralateral—42%, *p* = 0.02), and an increased α-2-macroglobulin activity on both sides (ipsilateral—53%, *p* = 0.03; contralateral—56%, *p* = 0.037) compared to controls. These changes are considered as potential biomarkers for differential diagnosis. Similar changes in the TF were found in 1-methyl-4-phenyl-1,2,3,6-tetrahydropyridine (MPTP)-treated mice when modeling clinical and preclinical stages of PD. These data show the adequacy of models to the pathogenesis of PD along the selected metabolic pathways, and also suggest that the found TF changes can be considered as potential biomarkers for preclinical diagnosis of PD. In Parkinsonian mice, the level of catecholamines also changes in the lacrimal glands, which makes it possible to consider them as one of the sources of catecholamines in the TF.

## 1. Introduction

One of the global issues in the XXI century is the fight against socially significant, still incurable neurodegenerative diseases. In this context, Parkinson’s disease (PD) holds second one in terms of incidence and severity. It is diagnosed by the manifestation of motor symptoms (tremor and bradykinesia) [1], which appear decades after the onset of pathological processes [2]. By that time, most of the nigrostriatal dopaminergic neurons, a key link in the regulation of motor function, have degenerated. This explains the limited efficacy of current symptomatic pharmacotherapy [3,4].

The main hope for increasing the efficacy of PD treatment is associated with the development of early (preclinical) diagnosis and preventive neuroprotective therapy. It is believed that neuroprotective therapy at the preclinical stage of PD should slow down the death of neurons and thereby significantly prolong the period of physical and social activity of patients [4,5,6]. The most widely used methodology for developing an early diagnosis of PD is based on the fact that this disease is systemic. In addition to the nigrostriatal dopaminergic system, pathological processes extend to other areas of the central and peripheral nervous system, which results in the impairment of sleep and olfaction, constipation, orthostatic hypotension, as well as changes in body fluids that can be used as diagnostic biomarkers [4,7,8,9,10,11,12,13].

The above systemic manifestations of PD in untreated patients at the early clinical stage are considered not only as biomarkers of the clinical stage, but also as potential biomarkers of the preclinical stage of PD. However, there are no evidence for this, and all biomarkers detected so far are non-specific [9,14]. Moreover, this paradigm does not take into account the fact that as pathological processes spread throughout the body and the disease progresses, the range of biomarkers should expand significantly.

To solve this problem, we have recently proposed the use of animal models of PD to validate blood changes found in patients as biomarkers of the preclinical stage of PD [4,14,15,16]. According to this methodology, the presence of common biomarkers in patients and in animals in a model of the early clinical stage indicates an adequate reproduction of the pathogenesis of PD, at least along this metabolic pathway. The detection of the same biomarker in animals in a model of the preclinical stage of PD suggests that it is also a characteristic of patients at the preclinical stage of the disease [14].

Until recently, the development of a differential and early diagnosis of PD has been based on the search for changes in the cerebrospinal fluid and blood in PD patients at an early clinical stage [4,17]. However, in recent years, the search for changes in the TF has also been considered promising for the development of the PD diagnosis [18,19,20]. The attractiveness of using the TF is explained by the fact that its collection is a non-invasive, easily reproducible procedure without negative consequences [20]. 

It has been shown that in the TF of PD patients, the levels of proteins associated with neurodegeneration change significantly. In fact, when doing proteomics [18], it was shown that in the TF of PD patients (*n* = 36) the levels of 21 proteins increase and the levels of 19 proteins decrease in comparison with the control (*n* = 18). More specifically, Çomoğlu [21] found in the TF a doubling of the TNF-α protein level, a biomarker of neuroinflammation, in a cohort of 17 PD patients compared with 17 control subjects, and Hamm-Alvarez [22] found in the TF of PD patients (84 PD patients vs. 84 controls) a 4-fold increase in α-synuclein, which in PD becomes toxic due to aggregation and phosphorylation [7,8]. Along with these proteins, α-2-macroglobulin, a protease inhibitor, which is also involved in the pathogenesis of PD, was found in human TF [23,24]. Moreover, the gene encoding this protein is considered as a PD risk gene [25,26,27]. It is noteworthy that in PD, the level of α-2-macroglobulin in the CSF changes, which is considered as a diagnostic marker [24,28,29]. Based on these data, one of the objectives of this study was to assess for the first time the activity of α-2-macroglobulin in the TF in PD patients.

According to our preliminary data obtained in a rather small cohort of patients [15], besides proteins the level of some catecholamines in the TF can change in PD, in addition to protein levels [15]. This is probably due to the fact that central and peripheral catecholaminergic neurons, degenerating in PD, play a key role not only in the regulation of motor function, but also in the regulation of the eye [30,31]. In fact, the eye, including the conjunctiva and cornea, and surrounding tissues have extensive sympathetic innervation [20,32,33], and catecholamines have been found in the TF [15,34,35,36]. This idea is supported by our previous observation of the degradation of the catecholaminergic systems of the eye in parkinsonian mice [37].

Although there are still no studies of catecholamines in TF in PD patients, numerous, though conflicting data were accumulated on a change in the level of catecholamines in the cerebrospinal fluid and blood in PD patients (*n* = 9–53), which are considered as potential diagnostic markers [38,39,40,41,42,43]. Based on the above, the next objective of this study was to definitely determine in the enlarged cohort of PD patients whether changes in catecholamine levels in the TF are characteristic of PD and could be considered as potential diagnostic biomarkers. Interestingly, catecholamines and α-2-macroglobulin, selected in this study as potential biomarkers in TF, can be functionally linked in PD. In fact, monoamine-activated α-2-macroglobulin can stimulate the release of dopamine in the striatum [44]. 

The third objective of this study was to compare TF changes in PD patients with those in animals in neurotoxic 1-methyl-4-phenyl-1,2,3,6-tetrahydropyridine (MPTP) models of the early clinical stage and preclinical stage of PD according to the methodology outlined above [16].

The last objective of this study was to test whether catecholamines are synthesized in the lacrimal glands in mice and their metabolism is changed when modeling PD. This might clarify if the lacrimal glands can be considered as one of the sources of catecholamines in the TF.

## 2. Results

### 2.1. Clinical and Ophtalmological Characteristics of PD Patients and Control Subjects

In this study, TF was obtained for analysis in 31 PD patients, male and female, at an early clinical stage (Hoehn and Yahr, stage 1–2) before starting antiparkinsonian therapy, as well as in 32 age-matching control subjects of both sexes. Main clinical characteristics of studied cohorts are presented in Table 1.

According to our data, the TF volume in the PD patients was lower than in controls by 30% (*p* = 0.011). However, there were no differences in intraocular pressure between the PD patients and the control group (Table 1). Considering that TF volume in PD patients differs from that in control subjects, we evaluated both the concentration and the content (concentration × tear fluid volume) of catecholamines and metabolites. Evaluation of the content makes it possible to be sure that the observed changes in concentration are due to a change in the secretion of the detected substances, and not only to a change in TF volume.

### 2.2. Quantification of Catecholamines and Metabolites and Activity of α-2-Macroglobulin in the Tear Fluid in PD Patients and the Control Group

**Figure A1:**
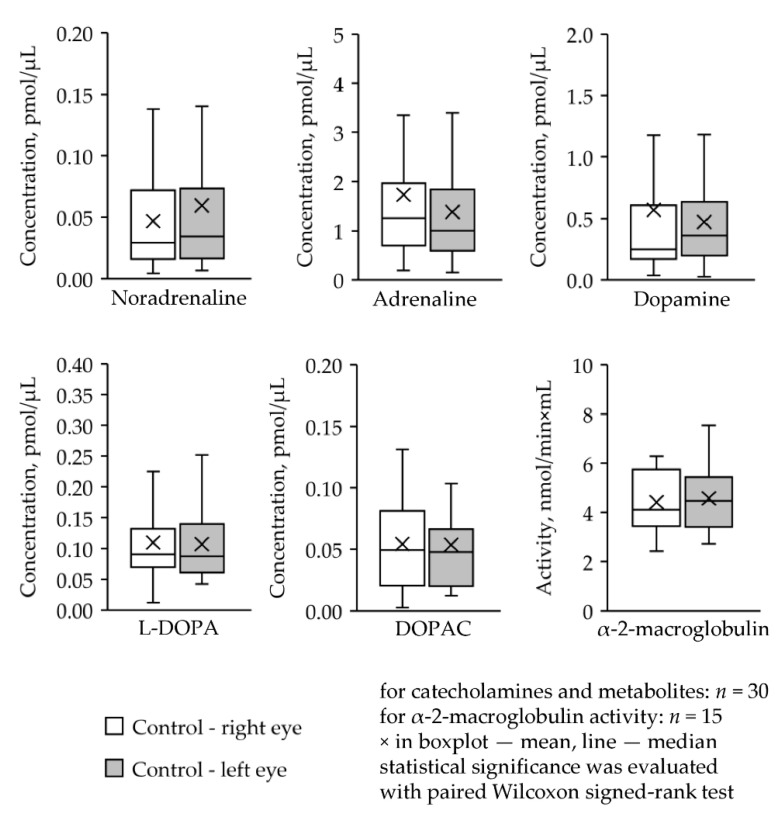
Concentration of catecholamines and metabolites and the activity of α-2-macroglobulin in the tear fluid collected from right and left eyes of control subjects. DOPAC, dihydroxyphenylacetic acid; L-DOPA, L-3,4-dihydroxyphenylalanine.

Catecholamines (dopamine, noradrenaline, and adrenaline) and some metabolites, L-3,4-dihydroxyphenylalanine (L-DOPA) (dopamine precursor) and dihydroxyphenylacetic acid (DOPAC) (product of dopamine degradation) were measured in the TF of PD patients and control subjects. Since PD develops asymmetrically [45], data on the content/concentration of catecholamines in TF in patients were assessed separately for the eye on the side of pronounced motor symptoms (ipsilateral side) and on the side of no or less pronounced motor symptoms (contralateral side). Since there is no asymmetry in the tear fluid composition in control subjects (Appendix A Figure A1), the control data were used as the average for the right and left eyes.

**Figure 1:**
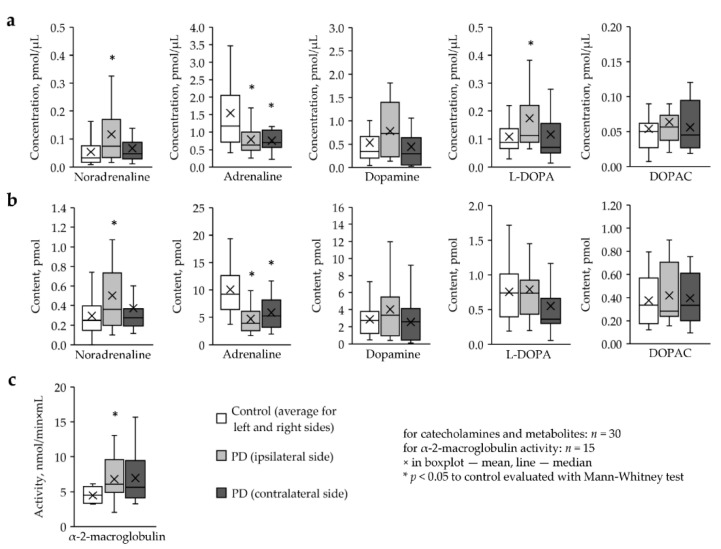
(**a**) Concentration and (**b**) content of catecholamines and metabolites and the activity of α-2-macroglobulin (**c**) in the tear fluid of PD patients on the ipsilateral and contralateral side and in control subjects. DOPAC, dihydroxyphenylacetic acid; L-DOPA, L-3,4-dihydroxyphenylalanine.

According to our data, the average concentration of noradrenaline in the TF of PD patients on the ipsilateral side was more than 2 times higher than in the control (0.12 pmol/μL versus 0.05 pmol/μL, *p* = 0.018, *n* = 30) (Figure 1a). The concentration of L-DOPA in PD patients on the ipsilateral side was 60% higher than in the control (0.17 pmol/μL versus 0.11 pmol/μL, *p* = 0.024, *n* = 30) (Figure 1a). On the contrary, the concentration of adrenaline in the TF of PD patients was approximately half as much on both sides as in the control group (ipsilateral PD—0.78 pmol/μL; contralateral PD—0.75 pmol/μL; control—1.54 pmol/μL; PD ipsilateral versus control: *p* = 0.023; PD contralateral versus control: *p* = 0.019, *n* = 30) (Figure 1a). No changes in dopamine and DOPAC concentrations were found in PD patients as compared to controls (Figure 1a).

When assessing the content of catecholamines and metabolites in the TF, we found an increase in the content of noradrenaline on the ipsilateral side (PD ipsilateral—0.50 pmol versus control—0.29 pmol, *p* = 0.049, *n* = 30) (Figure 1b), as well as a decrease in adrenaline content on both sides (PD ipsilateral—4.71 pmol, PD contralateral—5.84 pmol, control—10.08 pmol, PD ipsilateral versus control: *p* = 0.004, PD contralateral versus control: *p* = 0.02, *n* = 30) (Figure 1b). The content of dopamine, L-DOPA, and DOPAC in TF in PD remained at the control level (Figure 1b).

The activity of α-2-macroglobulin in TF in PD patients is increased by approximately 50% on both sides compared to control (PD ipsilateral—6.81 nmol/min×mL; PD contralateral—6.97 nmol/min×mL; control—4.46 nmol/min×mL; PD ipsilateral versus control: *p* = 0.031, PD contralateral versus control: *p* = 0.037, *n* = 15) (Figure 1c).

### 2.3. Comparison of Gender Differences in Biomarkers in the Tear Fluid of PD Patients and Controls and Correlation Analysis with the Severity of Disease 

We did not find gender differences in the concentration and content of catecholamines and metabolites, as well as in the activity of α-2-macroglobulin in the TF of PD patients and control subjects (Appendix A Table A1). When evaluating possible correlations between changes in TF collected in PD patients on the ipsilateral side and the severity of PD the only correlation was found between the concentration of dopamine and the patient status on the UPDRS III scale (Table 2).

### 2.4. ROC Analysis of Biomarkers in the Tear Fluid in PD Patients and Control Subjects

**Figure 2:**
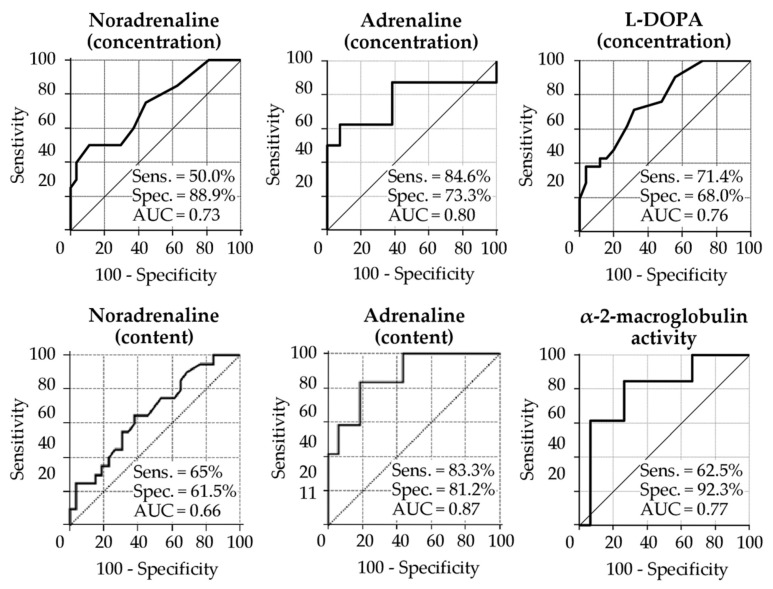
ROC curves of tear fluid biomarkers, collected on the ipsilateral side, for discriminating PD patients from controls. The area under the curve (AUC) and the percentage of sensitivity (Sens.) and specificity (Spec.) at cutoff points are indicated on each plot.

We used receiver operating characteristics (ROCs) to assess the diagnostic accuracy of markers found in the TF on the ipsilateral side of PD in this study. The area under the ROC curve (AUC) for potential biomarkers, which is represented by statistically significant changes in the concentration of noradrenaline, adrenaline, and L-DOPA, as well as the activity of α-2-macroglobulin, was 0.7 or higher. This means a good ability to discriminate between PD patients and control subjects (Figure 2). When using statistically significant changes in the content of catecholamines in the TF of PD patients for ROC analysis, the diagnostic efficacy for noradrenaline decreased (AUC = 0.66), and that for adrenaline increased (AUC = 0.87).

Analysis of the sensitivity and specificity at the selected cutoff points showed that concentration and content of adrenaline are characterized by the greatest sensitivity value (>80%), whereas noradrenaline concentration and α-2-macroglobulin activity show a very high specificity (>85%) (Figure 2).

### 2.5. Motor Behavior and Dopamine Levels in the Nigrostriatal System in MPTP-Treated and Control Mice

**Figure 3:**
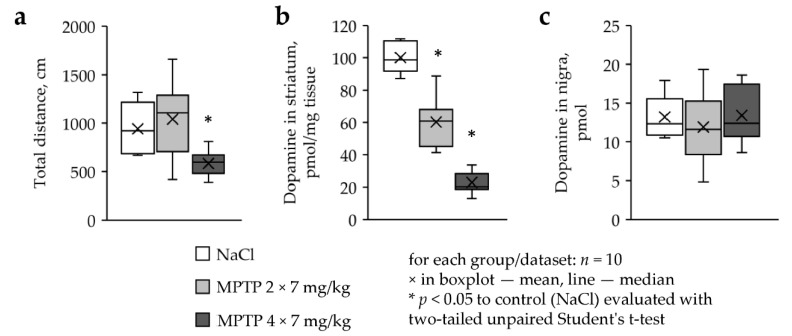
Behavioral and metabolic changes in mice two weeks following MPTP administration: (**a**) total distance in the open field test, (**b**) dopamine concentration in striatum, and (**c**) dopamine content in substantia nigra.

The distance traveled in the open field test did not change in mice treated with MPTP twice in a single dose of 7 mg/kg, compared with the control (Figure 3a). However, this parameter decreased by 44% in mice treated with MPTP four times in a single dose of 7 mg/kg (584 cm versus 943 cm, *p* = 0.002, *n* = 10). In the same experiments, the dopamine concentration in the striatum decreased by 40% and 77%, respectively (MPTP 2 × 7—60.3 pmol/mg, MPTP 4 × 7—22.9 pmol/mg, control—100.1 pmol/mg, MPTP 2 × 7 versus control: *p* = 0.001, MPTP 4 × 7 versus control: *p* = 0.0013, *n* = 10) (Figure 3b). In contrast to the striatum, there was no change in the dopamine content in the substantia nigra in both experiments (Figure 3c).

### 2.6. Concentration and Content of Catecholamines and Metabolites, as well as the Activity of α-2-Macroglobulin in the Tear Fluid in MPTP-Treated and Control Mice

We were able to evaluate noradrenaline, dopamine, and DOPAC in mice TF samples, whereas adrenaline and L-DOPA were undetectable in our assay. Considering that there is a tendency (*p* = 0.11) to increase the volume of TF in mice receiving four injections of MPTP at a single dose of 7 mg/kg, we also estimated, in addition to the concentration, the content of catecholamines and metabolites in TF in mice in both experiments (MPTP: 2 × 7 mg/kg or 4 × 7 mg/kg).

**Figure 4:**
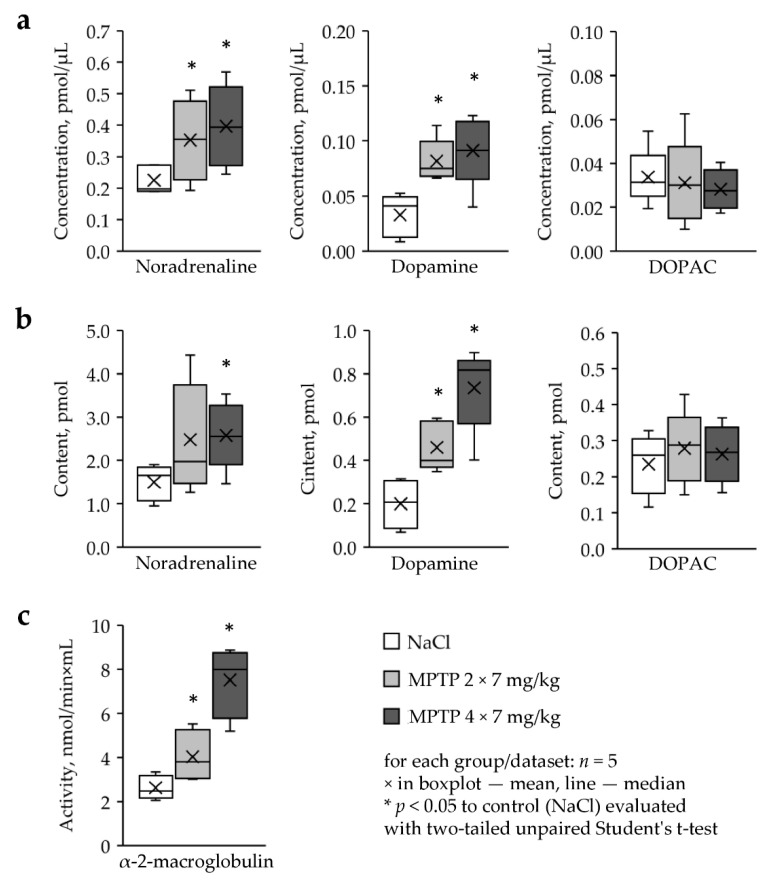
(**a**) Concentration and (**b**) content of catecholamines and metabolites, as well as (**c**) the activity of α-2-macroglobulin in the tear fluid in mice two weeks following MPTP administration. DOPAC, dihydroxyphenylacetic acid.

The concentration of noradrenaline in the TF in mice that received MPTP twice in a single dose of 7 mg/kg, increased by 56%, and in mice that received MPTP four times in the same single dose, the concentration rose by 76%, compared to the control (MPTP 2 × 7—0.35 pmol/L MPTP 4 × 7—0.4 pmol/µL, control—0.23 pmol/µL, MPTP 2 × 7 versus control: *p* = 0.036, MPTP 4 × 7 versus control: *p* = 0.024, *n* = 5) (Figure 4a). Moreover, in both experiments, we found an almost 3-fold increase in dopamine concentration in the TF compared to the control (MPTP 2 × 7—0.08 pmol/µL, MPTP 4 × 7—0.09 pmol/µL, control—0.03 pmol/µL, MPTP 2 × 7 versus control: *p* = 0.004, MPTP 4 × 7 versus control: *p* = 0.008, *n* = 5), whereas the DOPAC concentration did not change (Figure 4a).

From our analysis of the content of catecholamines and metabolites in the TF in mice it follows that noradrenaline content increased by 73% only in mice that received MPTP four times at a single dose of 7 mg/kg compared to control (2.58 pmol versus 1.49 pmol, *p* = 0.024, *n* = 5) (Figure 4b). The content of dopamine in the TF of MPTP-treated mice was also increased compared to the control (MPTP 2 × 7—0.46 pmol, MPTP 4 × 7—0.74 pmol, control—0.2 pmol, MPTP 2 × 7 vs. control: *p* = 0.012, MPTP 4 × 7 vs. control: *p* = 0.002, *n* = 5), whereas the DOPAC content did not change (Figure 4a).

The activity of α-2-macroglobulin increased by 53% in TF in mice receiving MPTP twice at a single dose of 7 mg/kg compared to the control, and it increased almost 3-fold in mice receiving MPTP four times at the same single dose (MPTP 2 × 7—4.04 nmol/min×ml, MPTP 4 × 7—7.51 nmol/min×mL, control—2.63 nmol/min×mL, MPTP 2 × 7 vs. control: *p* = 0.048, MPTP 4 × 7 vs. control: *p* = 0.0004, *n* = 5) (Figure 4c).

### 2.7. Concentration of Catecholamines in Lacrimal Glands in MPTP-Treated and Control Mice

**Figure 5:**
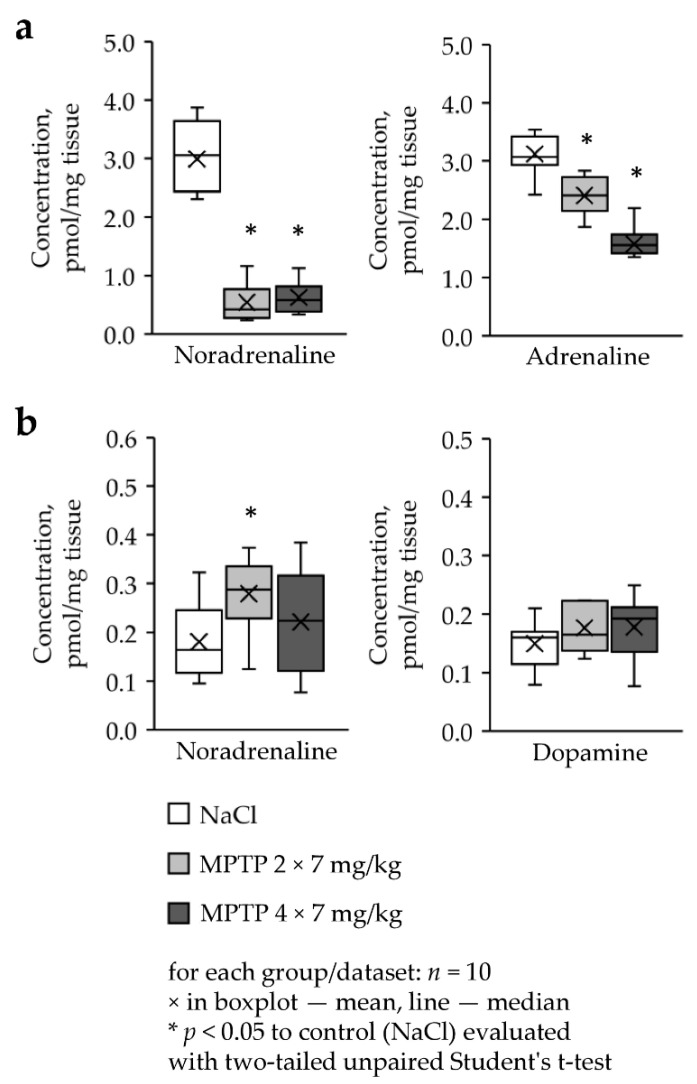
Concentration of catecholamines in the (**a**) lacrimal exorbital and **(b**) Harderian glands in mice two weeks following MPTP administration.

We were able to evaluate only noradrenaline and adrenaline in exorbital lacrimal glands of mice and noradrenaline and dopamine in Harderian glands (Figure 5). Other catecholamines and metabolites were undetectable in our assay.

The concentration of noradrenaline decreased approximately by 5 times in the exorbital lacrimal glands in mice after 2-fold and 4-fold administration of MPTP at a single dose of 7 mg/kg compared with control (MPTP 2 × 7—0.55 pmol/mg, MPTP 4 × 7—0.63 pmol/mg, control—2.99 pmol/mg, MPTP 2 × 7 versus control: *p* = 0.0003, MPTP 4 × 7 versus control: *p* = 0.0001, *n* = 10) (Figure 5a). The concentration of adrenaline also decreased in the exorbital lacrimal glands in mice after 2-fold and 4-fold administration of MPTP at a single dose of 7 mg/kg by 23% and 50%, respectively (MPTP 2 × 7—2.41 pmol/mg, MPTP 4 × 7—1.57 pmol/mg, control—3.12 pmol/mg, MPTP 2 × 7 versus control: *p* = 0.0004, MPTP 4 × 7 versus control: *p* = 0.00001, *n* = 10) (Figure 5a).

The concentration of noradrenaline in the Harderian glands of mice that received MPTP twice increased by 55% compared with the controls (0.28 pmol/mg versus 0.18 pmol/mg, *p* = 0.0241, *n* = 10) (Figure 5b). In mice that received four MPTP injections, the noradrenaline level in the Harderian glands did not change. Moreover, no change was found in the concentration of dopamine in mice receiving twice or four times MPTP (Figure 5b).

## 3. Discussion

### 3.1. Catecholamines and Metabolites in the Tear Fluid as Potential Biomarkers of PD

The main trend in the development of an early and differential diagnosis of PD is the search for biomarkers as changes in the level of certain substances in the body fluids, mainly in the blood and cerebrospinal fluid. However, data published by various authors are often contradictory [14,17,38,39,40,41,42,43,46,47,48]. This is probably due to the fact that the concentration of analytes in the blood and, to a lesser extent, in the cerebrospinal fluid is an integral index of a wide range of metabolic processes associated with degeneration and plasticity of central and peripheral neurons, as well as desympathization of the internal organs [8,20]. In this context, the use of the TF for searching PD biomarkers has certain advantages over the use of the cerebrospinal fluid and blood. These include non-invasive atraumatic collection and a smaller spectrum of substances contained in the TF [20,49]. 

Given that the eye, including the conjunctiva and cornea, which are one of the most important sources of substances in the TF, receives sympathetic innervation [20,50,51,52,53], we assumed that in PD associated with systemic degeneration of catecholaminergic neurons, the levels of catecholamines and their metabolites in the TF should change. However, considering the decrease in TF volume in PD patients, which was observed in this and previous studies [18,21,22], we have evaluated the change in the TF not only in the concentration, but also in the content of catecholamines and metabolites. The need for this is explained by the fact that a change in the concentration of certain substances in the TF of PD patients can be the result of a decrease in TF volume rather than a change in their secretion due to pathology. This idea was supported by the observation that, despite the difference in the concentration of L-DOPA in the TF in PD patients and the age-matched control observed in this study, the content of L-DOPA did not change (Table 3).

**Figure 6:**
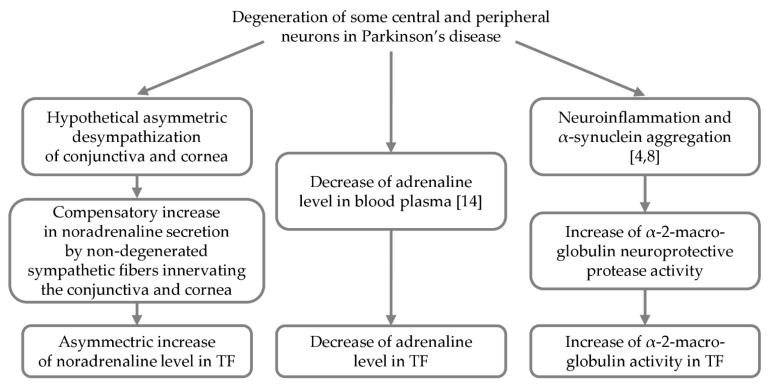
Schematic diagram of hypothetical mechanisms of observed changes in the tear fluid (TF).

In the TF of PD patients, but only on the side of pronounced motor disorders (ipsilateral side), we found an increase in the concentration and content of noradrenaline compared to the control (Table 3). Considering that adrenergic receptors are expressed in the cells of the conjunctiva and cornea facing the TF [54,55], an increase in the level of noradrenaline in the TF can be a compensatory mechanism under their desympathization associated with PD (Figure 6). In addition, we found a decrease in the concentration and content of adrenaline in the TF of PD patients, both in the ipsilateral and contralateral sides (Table 3). These data are difficult to interpret, although it is possible that adrenaline enters the TF from the blood (Figure 6), overcoming a blood-tear barrier, which becomes more permeable in pathology [56].

Data on the contrasting changes in the level of noradrenaline—an increase in the TF and a decrease in the plasma [14,15], respectively—attract special attention (Table 3). As for the interpretation of these data, one should proceed from the idea that the noradrenaline concentration in body fluids is an integral indicator of the complementary processes, neurodegeneration and neuroplasticity. This means that an increased level of noradrenaline in TF can suggest the local prevalence of compensatory processes over neurodegeneration (Figure 6). A decreased level of noradrenaline in the blood can be an indicator of the systemic prevalence of neurodegeneration over neuroplasticity.

We believe that changes in the levels of noradrenaline and adrenaline in the TF can be considered as potential biomarkers for a differential diagnosis of PD. It should be emphasized that we have shown, for the first time, significant differences in the level of noradrenaline and adrenaline, as well as in the α-2-macroglobulin activity in tears in untreated PD at the early clinical stage (Hoehn and Yahr, stages 1–2), despite great difficulties in selecting these patients.

### 3.2. Catecholamines and Metabolites in the Tear Fluid as Potential Biomarkers of PD at the Preclinical Stage

One of the objectives of this study was to determine whether changes in the content of catecholamines in the TF in untreated PD patients at an early clinical stage can be considered as potential diagnostic biomarkers of the preclinical stage. To address this issue, biomarkers found in the body fluids of patients have been validated in mice used for modeling PD at the preclinical and clinical stages MPTP. It should be noted that the neurotoxic effect of MPTP is similar to that of isoquinoline derivatives and paraquat, endogenous and exogenous neurotoxins, respectively, which cause the development of PD [57,58]. This new methodology was recently first applied to validation of the biomarkers of PD, catecholamines and amino acids, in the blood [14].

Despite the fact that the MPTP models of the preclinical and clinical stages of PD have been developed and used for ten years [16], they should be tested in every new study at least for motor behavior and dopamine content in the striatum. This is due to variations in the sensitivity to MPTP in mice of different generations [59]. In this study, the preclinical stage of PD was modeled in mice by a double subcutaneous injection of MPTP at a single dose of 7 mg/kg, which was lower than in our previous study (8 mg/kg) [15]. This model, in which the level of dopamine decreased by 40%, was better at reproducing the preclinical stage of PD, since in the previous study, the level of dopamine decreased by 65%, which is close to the threshold of 70%, at which there is a transition to the clinical stage, associated with the appearance of movement disorders. When modeling the early clinical stage of PD, mice were injected with MPTP 4 times at the same single dose and with the same interval between injections. This resulted in the threshold loss of dopamine (>70%) in the striatum and the appearance of motor disorders. 

For the assay of catecholamines, TF was pooled from two mice under short-term mild anesthesia. It follows from our results that in mice on both PD models, as in PD patients, the level of noradrenaline in the TF was increased as compared to the corresponding controls (Table 3). Given the unidirectional changes in the level of noradrenaline in the TF in PD patients and MPTP-treated mice, we believe that mild anesthesia did not significantly affect the mice. 

Based on the above methodology for validating biomarkers found in the body fluids of patients with the help of animal models, we believe that an increase in the level of noradrenaline in the TF in PD patients and animals used for modeling clinical and preclinical stages of PD can be considered as a potential biomarker of the preclinical stage. Indeed, a decrease in the level of noradrenaline in the TF of PD patients and in MPTP-treated mice at modeling an early clinical stage may indicate an adequate reproduction of the pathogenesis of PD, at least along this metabolic pathway. In turn, a decrease in the level of noradrenaline in the TF in the model of the PD preclinical stage allows us to consider this indicator as a potential diagnostic biomarker of the PD preclinical stage.

In contrast to noradrenaline, we were unable to validate in mice the change in the adrenaline content observed in the TF in PD patients, since adrenaline was undetectable in the TF in control and MPTP-treated mice (Table 3). Nevertheless, this weak point of our study does not exclude the possibility that a 2-fold decrease in the adrenaline content in the TF of PD patients can be considered biomarker for the preclinical stage of PD.

### 3.3. Activity of α-2-Macroglobulin in Tears as a Biomarker of PD

We have shown for the first time that in PD patients, the activity of α-2-macroglobulin is almost doubled. Interestingly, in contrast to the asymmetric change in the noradrenaline level in the TF, the change in α-2-macroglobulin activity is symmetrical (Figure 6). This is probably due to the fact that even with asymmetric degradation of catecholaminergic systems, at least of the nigrostriatal dopaminergic system [45], the pathogenesis of PD is characterized by general symmetric manifestations, such as neuroinflammation, circulation of aggregated α-synuclein, and neuroplasticity. α-2-macroglobulin is involved in the regulation of these asymmetric processes (Figure 6) [24]. A significant increase in the activity of α-2-macroglobulin in the TF was also shown in mice in the model of the preclinical stage of PD and, to an even greater extent, in the model of the clinical stage of PD. 

Given the wide variety of α-2-macroglobulin functions, it is difficult to unambiguously interpret the changes in its activity that we found in PD patients and in animal models of PD (Figure 6). Indeed, these changes can be the result of the α-2-macroglobulin ability to inhibit proteolytic enzymes, bind cytokines, growth factors, apolipoproteins, and other proteins, which can be associated with PD [60]. In the pathogenesis of neurodegenerative diseases and, in particular, PD, the neuroprotective chaperone activity of α-2-macroglobulin is of particular interest. In this case, α-2-macroglobulin serves to stabilize malformed proteins (β-amyloid and aggregated α-synuclein), preventing their aggregation and transformation into neurotoxins [24]. It should be noted that α-2-macroglobulin, in addition to neuroprotective processes, is involved in neurotoxic processes, e.g., suppressing the neuroprotective activity of nerve growth factor [61]. In any case, the data obtained in this study from PD patients and MPTP-treated mice suggest that the increased α-2-macroglobulin activity in the TF can be considered as a potential diagnostic biomarker for both clinical and preclinical stages of PD (Table 3).

### 3.4. Diagnostic Accuracy of Tear Fluid Biomarkers 

The ROC analysis was used in this study to assess the accuracy of biomarkers found in the TF of PD patients. It is considered that the diagnostic efficacy of the biomarker is higher as the AUC increases. The diagnostic value of a biomarker is considered high if its specificity exceeds 80% [1]. The AUC of TF biomarkers tested in this study (catecholamines, α-2-macroglobulins) ranged from 0.66 to 0.87. This index is slightly lower than the AUC for biomarkers in the plasma of PD patients [14], but higher than that for TF biomarkers calculated in previous studies [22,43]. The most promising TF biomarkers found in this study are changes in noradrenaline concentration (specificity = 88.9%, AUC = 0.73), adrenaline content (specificity = 81.2%, AUC = 0.87) and α-2-macroglobulin activity (specificity = 92.3%, AUC = 0.77). These biomarkers are characterized by the highest values of specificity and AUC.

The most effective, but challenging approach to validating changes in body fluids found in PD patients at the clinical stage and in animal models of clinical and prelcinical stages of PD as biomarkers of the preclinical stage is to search for these biomarkers in risk subjects at the prodromal stage of PD [4]. In accordance with this approach, a risk group is first created. This includes the elderly subjects without motor disorders, but with premotor symptoms, mainly with sleep, smell, and peristalsis impairments. Risk subjects who manifest the same changes in body fluids as patients at the clinical stage of PD and animals on models of clinical and preclinical stages of PD undergo an examination of the nigrostriatal dopaminergic system with positron emission tomography. Changes in body fluids detected in risk subjects with a failure of the nigrostriatal dopaminergic system are considered diagnostic biomarkers of PD at the preclinical stage. Recently, this approach was used to validate changes in the cerebrospinal fluid and blood as biomarkers of the preclinical stage of PD. However, so far this approach has not been used to validate potential biomarkers in tears [4]. We intend to overcome this weakness in future research.

### 3.5. Lacrimal Glands as Potential Sources of Catecholamines in the Tear Fluid

When searching for biomarkers of PD in body fluids, in terms of applied neuroscience, it is enough to detect changes in the level of any substance. In terms of fundamental neuroscience, it is important to understand which cells are the sources of biomarkers and which pathological processes lead to their appearance. The lacrimal glands, which receive sympathetic innervation from the upper cervical ganglia, are generally considered one of the sources of catecholamines in TF [20,33,54]. 

Since obtaining the lacrimal glands (biopsy) in humans is impossible for ethical reasons, we evaluated for the first time the change in the content of catecholamines in the lacrimal glands in mice using PD models. It was shown that in MPTP-treated and control mice the lacrimal glands are characterized by both similarities and differences in the content of catecholamines. The similarity lies in the fact that both glands contain noradrenaline. The differences are that only the exorbital gland, in addition to noradrenaline, contains adrenaline, and only the Harderian gland, in addition to noradrenaline, contains dopamine. We have shown for the first time that a change in the metabolism of catecholamines in the lacrimal glands in MPTP-treated mice is associated with a change in the content of catecholamines in the TF. This suggests that lacrimal glands can be one of the sources of catecholamines in the TF. 

Thus, our data suggest that changes in the level of catecholamines and the activity of α-2-macroglobulin in the TF can be considered as potential biomarkers for a differential and preclinical diagnosis of PD, and the lacrimal glands can be regarded as one of the possible sources of catecholamine in the TF.

## 4. Materials and Methods

### 4.1. Characteristics of PD Patients and Control Subjects Designed for Tear Fluid Sampling

PD was diagnosed by the definition of motor syndrome as bradykinesia in combination with resting tremor, rigidity, or both, as well as its unilateral manifestation in some patients, which met the Clinical Diagnostic Criteria for Parkinson’s Disease of the Movement Disorders Society [1]. Motor symptoms were additionally assessed using the United Parkinson’s Disease Rating Scales (UPDRS), parts II and III [62]. PD patients and control subjects selected by neurologists were examined by ophthalmologists. Patients and controls with ophthalmic diseases such as acute ocular inflammation, ocular trauma, and non-PD retinal diseases were excluded from the study. The clinical protocol was approved by the Ethics Committee of the Sechenov First Moscow State Medical University (protocol № 34-20, date of approval—9 December 2020). The main criteria for the inclusion and exclusion of patients and control subjects are presented in Table 4.

In the selected subjects (*n* = 31 for PD patients and *n* = 32 for controls), the intraocular pressure was first measured, and then the TF was collected using sterile filter paper (5 mm wide), which was placed behind the lower eyelid, as in the Schirmer test [18,21]. TF was collected for 5 min by spontaneous sorption on a test strip without stimulating lacrimation. The length of the wetted portion of the strip was measured to calculate the sample volume by comparison with standard volume samples. Then the strips were placed in tubes with 100 μL 0.1 N HClO_4_ (for the assessment of catecholamines) or without it (for the assessment of α-2-macroglobulin), frozen in liquid nitrogen, and stored at −70 °C. Samples collected from each eye were stored and analyzed separately.

### 4.2. Animals and Experimental Procedures 

We used 30 male mice C57Bl/6 at the age 2–2.5 months (22–26 g), purchased in the “Pushchino” SPF animal facility (Pushchino, Moscow Oblast, Russia). The animals were kept at 21–23 °C in a light–dark 12 h cycle at free access to food and tap water. PD at the clinical and preclinical stages was modeled in mice by subcutaneous administration of MPTP (Sigma-Aldrich, St. Louis, MO, USA), twice at the individual dose of 7 mg/kg (*n* = 10) or four times at the individual dose of 7 mg/kg (*n* = 10), respectively, with a 2 h interval between injections. The control groups of animals received 0.9% NaCl instead of MPTP three times (*n* = 10). Two weeks after the administration of MPTP or 0.9% NaCl, the motor behavior of the mice was assessed in the open field test for 6 min using a PhenoMaster device for analyzing animal behavior (TSE Systems, Bad-Homburg, Germany).

Experimental procedures were carried out in accordance with the NIH Guide for the Care and Use of Laboratory Animals and were approved by the Animal Care and Use Committee of the Koltzov Institute of Developmental Biology RAS (protocol № 38-20, date of approval—30 July 2020).

### 4.3. Tear Fluid and Brain Tissue Sampling in Animals

To obtain TF, the animals were lightly anesthetized with isoflurane. Then the TF was collected using strips of filter paper 2.5 mm wide, similar to Schirmer’s strips, for 3 min without stimulating lacrimation. Lacrimal strips pooled from two mice served as one sample for analysis. These strips were placed in tubes with or without 0.1 N HClO_4_, frozen in liquid nitrogen and stored at −70 °C. 

After collecting the TF, the mice were decapitated, followed by dissection of the dorsal striatum and substantia nigra. Samples were weighed, frozen in liquid nitrogen, and stored at −70 °C. In addition to the striatum and substantia nigra, exorbital and Harderian lacrimal glands were collected. To dissect exorbital glands, the scalp was cut behind the lower jaw and ventrally from the auricle. Thereafter, the glands were removed through the above-mentioned incision as previously described [63,64]. Harderian glands were collected from the eye sockets after preliminary removal of the eyeballs and transection of the optic nerve. Lacrimal gland samples were weighed, frozen in liquid nitrogen, and stored at −70 °C.

### 4.4. HPLC Assay of Catecholamines and Metabolites

The content/concentration of catecholamines and metabolites (noradrenaline, adrenaline, dopamine, L-DOPA, and DOPAC) was measured using high performance liquid chromatography with electrochemical detection (HPLC-ED). First, samples of TF dissolved in perchloric acid were thawed. Then the eluate was taken and 3,4-dihydroxybenzylamine (DHBA)(Sigma-Aldrich, St. Louis, MO, USA), the internal standard, was added to a final concentration of 25 pmol/mL. Tissue samples were homogenized using a Labsonic M ultrasonic homogenizer (Sartorius, Goettingen, Germany) in 200 μL 0.1 N. HClO_4_ with 25 pmol/mL DHBA, followed by centrifugation at 2000× *g* for 20 min. In the prepared samples, catecholamines and metabolites were measured by HPLC, as previously described [15].

### 4.5. In Vitro Assay of α-2-Macroglobulin Activity

The estimation of α-2-macroglobulin activity was based on the fact that the complex of α-2-macroglobulin with trypsin retains proteolytic activity for the low-molecular-weight substrate N-benzoyl-DL-arginine-p-nitroanilide, and this activity is not affected by the soybean trypsin inhibitor. The α-2-macroglobulin activity was measured on a Synergy MX microplate photometer (BioTek, Winooski, VT, USA). Then it was calculated using a calibration curve plotted against p-nitroaniline (range: 0–28 nmol) and expressed in nmol/min per mL of the sample as previously described [65].

### 4.6. Reproducibility of the Assays

To increase the reproducibility of the HPLC assay and to compensate for possible inter-assay variations, we used the internal standard DHBA. Intra-assay coefficients of variation (CV) calculated based on DHBA peak area in each sample were less than 5% for tissue samples (for the reported assay of striatum CV = 0.96%, for nigra CV = 2.1%, *n* = 10 for each assay), and less than 10% for TF samples (for mice TF CV = 9.41%, *n* = 15, for human TF CV = 9.82%, *n* = 42). Day-to-day variability of DHBA standard peak was characterized by CV = 2.84% (*n* = 5). The lower limit of detection for catecholamines and metabolites was 5 pmol/mL (5:1, S:N). Calibration curves with DHBA were evaluated in blank samples (r^2^ = 0.9999), striatum homogenate (r^2^ = 0.9953) and human TF (r^2^ = 0.9999), which means no significant matrix effect.

For the reported assay of α-2-macroglobulin activity, intra-assay CV calculated based on standard concentrations of p-nitroaniline was 2.25% (*n* = 5), day-to-day variability was 2.5% (*n* = 5). The calibration curve was characterized by a correlation coefficient of r = 1 (*p* = 0.00005, *n* = 8, evaluated with Spearman’s test) meaning it was linear on the selected range.

### 4.7. Statistical Analysis 

Data are presented as group mean ± standard error of the mean. Data normality was checked using the Shapiro–Wilk test. Unpaired parametric data were processed using the unpaired Student’s t-test, and unpaired nonparametric data were processed using the Mann–Whitney test. Paired analysis was performed using the Wilcoxon signed-rank test. Correlation of TF biomarker levels in PD patients with Hoehn and Yahr and UPDRS scale scores was assessed by the Spearman’s test. Moreover, an analysis of the receiver operating characteristic (ROC) was performed to assess the diagnostic accuracy of biomarkers found in PD patients. Data from the ipsilateral side for the PD group and average of the left and right side values for the control was used. The optimal cutoff values of sensitivity and specificity were determined using the Youden index.

In all tests, p-values were two-tailed, and *p* < 0.05 was considered statistically significant. Analyses were performed using GraphPad Prism 6.0 (GraphPad Software, San Diego, CA, USA).

## 5. Conclusions

The following important results were obtained in this study:

(1) Untreated PD patients at an early clinical stage manifest increased levels of noradrenaline and α-2-macroglobulin activity, as well as a decrease in the level of adrenaline in the TF, which are considered as candidate biomarkers for the differential diagnosis of PD;
(2) Changes in the level of noradrenaline were found in the TF of PD patients mainly on the ipsilateral side, whereas changes in the level of adrenaline and α-2-macroglobulin activity were observed in TF on both sides;
(3) Changes in the level of noradrenaline and α-2-macroglobulin activity in TF observed in untreated PD patients at the clinical stage have been validated in animal models of PD as potential biomarkers of the preclinical stage;
(4) The lacrimal glands in control animals and in PD models contain catecholamines; their concentration in control animals differs from that in PD models, which suggests that these glands are one of the sources of catecholamines in TF.

## Figures and Tables

**Table 1:** Clinical evaluation, tear fluid volume and intraocular pressure in PD patients and controls.

	Cohort	Early Symptomatic Untreated PD Patients	Control Subjects
Parameter			
N		31	32
Gender, male/female		18/13	11/21
Age, years		60.4 ± 1.9	56.6 ± 2.0
PD stage assessment	Hoehn–Yahr scale	1.7 ± 0.1	–
	UPDRS II (daily activity)	8.0 ± 0.9	–
	UPDRS III (motor activity)	22.8 ± 2.2	–
Motor symptoms	duration since onset, years	2.4 ± 0.3	–
	side of onset, right/left	15/16	–
Total volume of tear fluid collected from both eyes, µL		11.7 ± 0.9 *	16.8 ± 1.7
Intraocular pressure in right/left eye, mm hg		23.6 ± 0.8/23.7 ± 0.7	22.1 ± 0.7/22.5 ± 0.7

* *p* < 0.05 to control, evaluated with two-tailed unpaired Student’s *t*-test.

**Table 2:** Correlation analysis of changes in the composition of the tear fluid in PD patients on the ipsilateral side and the patient status according to the Hoehn and Yahr and UPDRS III scales.

	Parameter 1	Hoehn and Yahr Score (Range: 1–3)		UPDRS III Score (Range: 4–36)	
Parameter 2		r	*p*-Value	r	*p*-Value
Concentration	Noradrenaline	0.33	0.15	0.36	0.12
	L-DOPA	0.17	0.45	0.28	0.22
	DOPAC	0.00	1.00	−0.02	0.97
	Dopamine	0.31	0.20	0.50	0.04 *
	Adrenaline	0.35	0.24	0.37	0.22
Content	Noradrenaline	0.31	0.18	0.21	0.37
	L-DOPA	−0.01	0.96	−0.04	0.85
	DOPAC	−0.02	0.94	−0.02	0.95
	Dopamine	0.18	0.44	0.06	0.81
	Adrenaline	−0.38	0.22	−0.50	0.09
α-2-macroglobulin activity		−0.12	0.78	−0.33	0.39

* *p* < 0.05, evaluated with two-tailed Spearman’s test; *n* = 31 for concentration and content of catecholamines; *n* = 15 for the α-2-macroglobulin activity UPDRS, Unified Parkinson’s Disease Rating Scale.

**Table 3:** Comparison of the level of catecholamines and the activity of α-2-macroglobulin in the tear fluid in PD patients and/or in mice treated with MPTP with the corresponding parameters in controls.

	**Source**	**Tear Fluid**				**Exorbital ** **Glands**		**Blood Plasma ** **(from [14]** **)**		
**Biomarker**		PD patients, ipsilateral side	PD patients, contralateral side	Mice, modeling preclinical PD	Mice, modeling clinical PD	Mice, modeling preclinical PD	Mice, modeling clinical PD	PD patients	Mice, modeling preclinical PD	Mice, modeling clinical PD
Concentration/content	Noradrenaline	↑/↑	=/=	↑/=	↑/↑	↓	↓	↓	=	=
	Adrenaline	↓/↓	↓/↓	-	-	↓	↓	↓	=	=
	Dopamine	=/=	=/=	↑/↑	↑/↑	-	-	↓	↓	=
	L-DOPA	↑/=	=/=	-	-	-	-	↓	↓	↓
	DOPAC	=/=	=/=	=/=	=/=	-	-	↓	↓	↓
α-2-macroglobulin activity		↑	↑	↑	↑	n/s	n/s	n/s	n/s	n/s

↑, increased compared to control; ↓, decreased compared to control; =, no difference compared to control; -, undetectable; n/s—non-studied.

**Table 4:** Inclusion and exclusion criteria for the selection of PD patients and control subjects.

№	Criteria	PD Patients	Control
1.	Idiopathic PD	*+*	*-*
2.	Secondary Parkinsonism	*-*	*-*
3.	Other extrapyramidal and neurological diseases	*-*	*-*
4.	Psychiatric disorders	*-*	*-*
5.	Ophthalmic diseases (acute eye inflammation, ocular trauma, PD related retinal disorders)	*-*	*-*
6.	Endocrine diseases	-	-
7.	Stroke and trauma over the past two years	-	*-*
8.	Somatic symptom disorders	*-*	*-*
9.	Neoplasms, including malignant tumors	*-*	*-*
10.	Specific antiparkinsonian therapy (levodopa, dopamine receptor agonists, monoamine oxidase inhibitors, amantadine, etc.)	*-*	*-*
11.	Antagonists of dopamine receptors (metoclopramide, domperidone, cinnarizine, etc.)	-	-
12.	Sympatholytics (reserpine)	*-*	*-*
13.	Agonists and antagonists of serotonin and adenosine receptors	*-*	*-*

+, inclusion criterion; -, exclusion criterion.

## Data Availability

Raw data could be provided from authors upon reasonable request.

## Appendix A

**Table A1:** The concentration and content of catecholamines and metabolites, as well as α-2-macroglobulin activity in the tear fluid in control subjects and PD patients, males and females.

	Control	Right Eye			Left Eye		
Biomarker		Male	Female	*p*-Value	Male	Female	*p*-Value
Concentration, pmol/µL	Noradrenaline	0.03 ± 0.01	0.05 ± 0.01	0.31	0.04 ± 0.01	0.05 ± 0.01	0.27
	L-DOPA	0.09 ± 0.02	0.10 ± 0.01	0.69	0.10 ± 0.02	0.09 ± 0.01	0.69
	DOPAC	0.05 ± 0.03	0.06 ± 0.01	0.66	0.05 ± 0.03	0.06 ± 0.01	0.81
	Dopamine	0.64 ± 0.30	0.53 ± 0.16	0.71	0.57 ± 0.11	0.42 ± 0.08	0.26
	Adrenaline	1.44 ± 0.59	2.10 ± 0.50	0.45	1.15 ± 0.25	1.86 ± 0.39	0.27
Content, pmol	Noradrenaline	0.23 ± 0.06	0.31 ± 0.05	0.29	0.23 ± 0.06	0.36 ± 0.08	0.28
	L-DOPA	0.89 ± 0.13	0.77 ± 0.10	0.48	0.59 ± 0.09	0.73 ± 0.10	0.37
	DOPAC	0.41 ± 0.20	0.36 ± 0.07	0.76	0.55 ± 0.14	0.33 ± 0.08	0.19
	Dopamine	3.11 ± 0.83	2.75 ± 0.47	0.69	2.63 ± 0.38	2.68 ± 0.60	0.95
	Adrenaline	11.2 ± 1.99	10.7 ± 1.84	0.88	9.84 ± 2.04	9.35 ± 1.50	0.88
α-2-macroglobulin activity, nmol/min×mL		5.16 ± 0.58	4.19 ± 0.33	0.16	4.92 ± 0.80	4.37 ± 0.44	0.53
	**PD Patients**	**Ipsilateral Side**			**Contralateral Side**		
**Biomarker**		**Male**	**Female**	***p*** **-Value**	**Male**	**Female**	***p*** **-Value**
Concentration, pmol/µL	Noradrenaline	0.10 ± 0.03	0.15 ± 0.05	0.39	0.08 ± 0.02	0.06 ± 0.01	0.56
	L-DOPA	0.18 ± 0.03	0.17 ± 0.05	0.93	0.14 ± 0.03	0.09 ± 0.02	0.29
	DOPAC	0.07 ± 0.02	0.04 ± 0.01	0.30	0.06 ± 0.01	0.06 ± 0.03	0.95
	Dopamine	0.71 ± 0.18	0.85 ± 0.22	0.63	0.54 ± 0.17	0.31 ± 0.08	0.31
	Adrenaline	0.73 ± 0.09	0.85 ± 0.25	0.60	0.84 ± 0.11	0.63 ± 0.19	0.31
Content, pmol	Noradrenaline	0.51 ± 0.14	0.49 ± 0.15	0.92	0.40 ± 0.10	0.33 ± 0.08	0.61
	L-DOPA	0.86 ± 0.13	0.70 ± 0.11	0.38	0.62 ± 0.13	0.48 ± 0.09	0.40
	DOPAC	0.42 ± 0.11	0.41 ± 0.11	0.92	0.38 ± 0.09	0.42 ± 0.14	0.81
	Dopamine	4.84 ± 1.45	3.01 ± 0.75	0.31	2.35 ± 0.58	2.84 ± 0.94	0.64
	Adrenaline	4.55 ± 1.04	4.93 ± 1.27	0.82	6.29 ± 1.30	5.21 ± 1.05	0.56
α-2-macroglobulin activity, nmol/min×mL		7.33 ± 1.46	5.96 ± 1.51	0.63	6.05 ± 1.18	8.49 ± 2.70	0.46

*p*-values are evaluated with Mann-Whitney test. For catecholamines and metabolites assay in control group *n* (male/female) = 11/21; in PD patients group *n* (male/female) = 18/13. For α-2-macroglobulin assay in control group: *n* (male/female) = 5/10; in PD patients group: *n* (male/female) = 6/9. DOPAC, dihydroxyphenylacetic acid; L-DOPA, L-3,4-dihydroxyphenylalanine.
